# Perfectionism and compulsive exercise: a systematic review and preliminary meta-analysis

**DOI:** 10.1007/s40519-024-01704-1

**Published:** 2025-01-16

**Authors:** Elizabeth Bills, Shannon R. Muir, Rose Stackpole, Sarah J. Egan

**Affiliations:** 1 School of Arts and Humanities, Edith Cowan University, Perth, Australia; 2 School of Population Health, Curtin University, Perth, Australia; 3 enAble Institute, Curtin University, Perth, Australia

**Keywords:** Perfectionism, Compulsive exercise, Eating disorder, Meta-analysis

## Abstract

**Purpose:**

There is a consistent link between perfectionism and compulsive exercise, and both are implicated in the maintenance of eating disorders, however no meta-analysis to date has quantified this relationship. We hypothesised that there would be significant, small-moderate pooled correlations between perfectionism dimensions and compulsive exercise.

**Methods:**

Published, peer-reviewed articles with standardised measures of perfectionism and the Compulsive Exercise Test were included. There were 7 studies included (*N* = 3117 participants, *M* age = 21.78 years, 49% female).

**Results:**

Total perfectionism (*r* = 0.37), perfectionistic strivings (*r* = 0.33), and perfectionistic concerns (*r* = 0.32) had significant pooled positive associations with compulsive exercise. Most studies (67%) were rated as fair or good quality as an indication of risk of bias. Limitations included the low number of available studies, the inclusion of only one clinical sample, and predominately cross-sectional studies which precluded causal inference.

**Conclusion:**

Higher perfectionism was associated with higher compulsive exercise. More research is needed on compulsive exercise to determine the best intervention approach given its relationship to perfectionism and relevance in the context of eating disorders.

**Level of evidence:**

Level I: Evidence obtained from a systematic review and meta-analysis.

## Introduction

Compulsive exercise is considered the continual rigid and extreme urge to exercise despite adverse consequences [1] and is strongly associated with eating disorders [2]. Compulsive exercise is identified in up to 80% of adults and 85% of adolescents with an eating disorder [3, 4]. It is associated with more severe eating disorder psychopathology, poorer quality of life, and elevated symptoms of depression and anxiety [5]. Compulsive exercise is linked to adverse treatment outcomes for individuals with eating disorders including lower remission rates [6], longer hospitalisations [7], relapse [8], and suicidal behaviour [9].

Compulsive exercise is also associated with eating disorder symptoms in non-clinical samples of adolescents [10]. Compulsive exercise has been associated with lower emotional regulation and quality of life, and greater psychological distress in non-clinical samples of adolescents [11, 12]. Hence, it is important to understand factors associated with compulsive exercise and eating disorder symptoms in both clinical and non-clinical samples to inform prevention and early intervention approaches.

Given the adverse outcomes associated with compulsive exercise in individuals with eating disorders, research is required to investigate constructs associated with compulsive exercise to improve understanding of its role in eating disorder symptoms. One construct shown to have a strong association with compulsive exercise is perfectionism [13] Perfectionism is a risk factor for the onset of compulsive exercise in adolescents [11] and is associated with poorer treatment outcomes [14]. In an inpatient sample with anorexia nervosa, those with high perfectionism compared to low perfectionism, have been demonstrated to have greater eating-related psychopathology, depressive symptoms, anxious temperament, and lower self-esteem [15].

Perfectionism has been defined as a multidimensional construct; factor analyses have identified two dimensions: perfectionistic strivings, characterised by setting high personal standards, and perfectionistic concerns, consisting of socially prescribed perfectionism, which refers to the perception that others expect high standards, and concern over mistakes [16, 17]. Despite meta-analyses demonstrating the association between perfectionism and eating disorders in adolescents [18] and adults [19], there has been no meta-analysis to date examining the relationship between compulsive exercise and perfectionism.

Meyer and colleagues’ [1] cognitive behavioural model of compulsive exercise identified reasons compulsive exercise may be present, in addition to eating disorders, including perfectionism, rigid personality traits, and using exercise as a means of affect regulation. It was theorised that perfectionistic individuals set unrelenting high standards in the areas of health, lifestyle, exercise, and sports [1]. Meyer et al. [1] proposed that there is a bidirectional pathway between eating disorder symptoms and compulsive exercise; perfectionism is theorised to maintain both compulsive exercise and eating disorder symptoms with perfectionism maintaining eating disorder symptoms indirectly through compulsive exercise. In this model, compulsive exercise and eating disorder symptoms compound as the personal and social benefits of losing weight reinforce a focus on eating, shape, weight, and their over-evaluation, leading to greater efforts towards dietary restraint [1]. Research in clinical and nonclinical populations supports Meyer and colleagues’ [1] model with findings that compulsive exercise had a direct effect on eating disorder symptoms [20, 21] and mediated the relationship between perfectionism and eating disorder symptoms [20]. Furthermore, perfectionism exhibited an indirect relationship, with eating disorder symptoms mediated by compulsive exercise, and compulsive exercise mediated by eating disorder symptoms [21]. Treatment targeted at reducing compulsive exercise has been associated with significant decreases in eating disorder symptoms [22] and perfectionism [23].

Despite this research, the literature regarding how to define and measure compulsive exercise is inconsistent and has resulted in a plethora of terms and measures [1]. Sicilia and colleagues’ [24] review of 17 different ‘problematic’ exercise screening measures indicated large theoretical differences between measures. These ranged from definitions of problematic exercise in terms of addiction and dependence (e.g., [25]), to weight control behaviour maintained by weight and shape concerns (e.g., [26]). Meyer and colleagues' [1] argued that many measures of exercise in eating disorder populations are too simplistic and fail to capture the multi-dimensional nature of compulsive exercise. The Compulsive Exercise Test (CET; [27]) is based on Meyer and colleagues’ cognitive behavioural model [1]. Compared to other measures of compulsive exercise, the CET [27] demonstrated a stronger association with eating disorder symptoms [28], was shown to be better at predicting eating disorder symptoms [29], and explained greater variance in eating disorder pathology [27]. The reliability and validity of the CET has been supported in research in clinical populations [30–32] and non-clinical populations (e.g., [10, 11, 33]).

Despite the consensus on the relevance of compulsive exercise and perfectionism to understanding symptoms of eating disorders, no meta-analysis has synthesised the relationship between compulsive exercise and perfectionism. Prior systematic reviews [34–36] have examined the association between perfectionism and exercise addiction. Exercise addiction is classified as a behavioral addiction and is a different construct to compulsive exercise in terms of affect regulation and its developmental sequence in relation to eating disorders [1]. The reviews of exercise addiction had conflicting conclusions; Çakın and colleagues [35] questioned the clinical relevance of the association between perfectionism and exercise addiction while González-Hernández and colleagues’ [36] review suggested a strong relationship between the two constructs. Two of the three systematic reviews described a stronger relationship between perfectionistic concerns and exercise addiction [34, 36]. The plethora of terms and use of multiple measures in the systematic reviews [34–36] means heterogeneity was a concern [36], which weakens the validity of these reviews [28].

The aim of this systematic review and meta-analysis is to investigate the association between compulsive exercise, as measured by the Compulsive Exercise Test, and total perfectionism, perfectionistic strivings, and perfectionistic concerns, in clinical and non-clinical samples. While perfectionism has been particularly associated with restrictive eating psychopathology in anorexia nervosa in some studies [15], we were interested in all eating disorder (ED) diagnoses, given reviews that have indicated the association between perfectionism and a range of ED diagnoses [22, 23].

Based on previous research [13], it is hypothesised that there will be a small positive pooled correlation between total perfectionism and compulsive exercise. Further, it is predicted that perfectionistic strivings will show a small positive pooled correlation, and perfectionistic concerns will show a moderate positive pooled correlation with compulsive exercise.

## Methods

The conduct and reporting of the systematic review and meta-analysis was based on the Preferred Reporting Items for Systematic Reviews and Meta-Analyses (PRISMA) statement [37] and the Cochrane Handbook [38]. The research protocol was pre-registered with the PROSPERO database on 2 September 2023 (approval number CRD42023459911, protocol available at: PROSPERO (york.ac.uk).

### Databases and search strategy

A literature search was completed on January 25, 2024, using the Ovid platform. The following databases were searched: Embase, Medline, PsychINFO, PsycARTICLES, Proquest, Scopus, and Web of Science. The search terms were perfectionism (searched as perfectionis*) and compulsive exercise (searched as [problematic or morbid or addiction or compulsive or dependence or excessive or obligatory or unhealthy or driven or rigid or abuse or patholog*] within two words proximity indicator [exercise or “physical activity” or training or running] or [“compulsive exercise test” or overexercis* or over-exercis* or overtraining]) (see Table 1). 

### Inclusion and exclusion criteria

The systematic review and meta-analysis inclusion criteria were: (a) published, peer-reviewed, quantitative research; (b) in English; (c) included a standardised measure of perfectionism and the Compulsive Exercise Test (CET; [27]); (d) reported unadjusted effect sizes; (e) provided adequate detail in the study to calculate effect sizes; and (f) were correlational study designs. Previous research has validated the utility of the three-factor model of the CET [39, 40]; hence both the three and five factor CET measures were included. We included both clinical eating disorder and non-clinical samples. In the clinical samples, any eating disorders defined in the Diagnostic and Statistical Manual of Mental Disorders (DSM-5) were eligible for classification as a clinical sample.

### Data collection and screening process

Articles were identified, screened, and assessed by the first author EB. Identified articles were downloaded into EndNote (Version 20.6) and uploaded to Research Screener, a validated semi-automated abstract screening tool [41]. EB was trained by SE in screening and meta-analysis procedures and screened at title and abstract level and then at full text level against the eligibility criteria. An independent reviewer RS, a PhD psychology student who had previously been trained and published a meta-analysis on eating disorders [19] with the senior author (SE), screened 100% (*n* = 113) of studies at abstract and full-text level. Inter-rater reliability via Cohen’s Kappa was calculated to show the level of agreement at both levels of screening [42]. Estimates of inter-rater reliability showed substantial agreement at title and abstract level (90% agreement; *k* = 0.63, 95% CI [0.46, 0.81]) and 100% agreement at full-text level [42]. Discrepancies were discussed with SE until a consensus was reached.

**Fig. 1:**
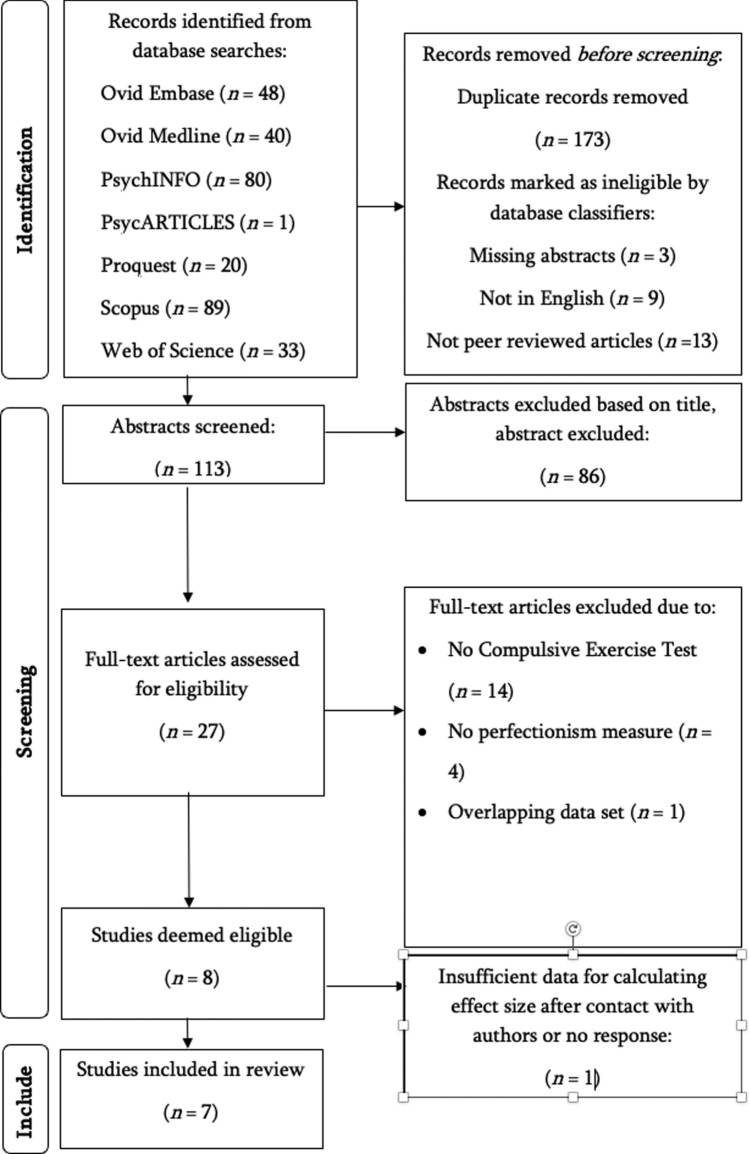
Process of study selection

One full text article could not be accessed. An email was sent to the author who responded with a copy of the article [43]. Additional data were requested from five authors who included measures of perfectionism and compulsive exercise but did not report a baseline correlation and additional data was obtained from four authors. Figure 1 outlines the PRISMA flow-chart including the identification, screening, eligibility, and exclusion process.

### Data extraction and management

The following data were extracted: author; publication year; country of origin; study design; sample size; population (i.e., clinical or non-clinical); mean age of the sample including standard deviation and range; percentage of female participants; instrument used to measure perfectionism; effect size between total perfectionism and compulsive exercise; effect size between perfectionistic strivings and compulsive exercise; and effect size between perfectionistic concerns and compulsive exercise.

### Classification of clinical and non-clinical eating disorder samples

Only one study was coded as clinical as the sample met criteria for an eating disorder diagnosis based on the Diagnostic and Statistical Manual [44]. The remaining studies were coded as non-clinical.

### Classification of perfectionistic strivings and concerns

To analyse the dimensions of perfectionism, studies were classified as measuring total perfectionism, perfectionistic strivings, and/or perfectionistic concerns. The subscale classifications of perfectionism measures were based on a meta-analysis by Bills et al. [18] of perfectionism and eating disorders (see Table 2). Two additional measures that were not classified in the review by Bills et al. [18] were included: the Spanish adaption of the Frost-Multidimensional Perfectionism Scale (FMPS; [45, 46]) and the Multidimensional Inventory of Perfectionism in Sport (MIPS; [47]). The Spanish FMPS subscales which uses four factors was classified into perfectionistic strivings and concerns based on prior studies (e.g., [48]). The MIPS comprises four subscales; only Striving for Perfection capturing perfectionistic strivings and Negative Reactions to Imperfections capturing perfectionistic concerns were included [49]. The subscale classifications of perfectionism measures are outlined in Table 2.

### Data analysis

Pearson’s *r* correlations were directly extracted from articles to examine the relationship between perfectionism and compulsive exercise. Effect sizes were interpreted according to Cohen’s [50] conventions (i.e., *r* = 0.10 small, *r* = 0.30 moderate, *r* = 0.50 large). The standard error of each effect size was calculated using the recommended calculation; SE = (1–*r*^2^) √ *N*–3 [51].

### Independence of effect sizes

For valid comparisons across studies where there were overlapping data sets, the most recent study was included, excluding one study [52]. To minimise potential effects of dependent data, where a study reported more than one perfectionism score, only one association was included and preference was given to the total perfectionism score over the perfectionistic concerns score [20]. Likewise, where studies reported multiple CET subscale correlations, the average effect size was calculated [20, 53].

### Quality assessment of risk of bias

The National Institute of Health’s [54] Quality Assessment Tool for Observational and Cross-Sectional Studies was used to assess the methodological quality of studies to indicate risk of bias. Study quality was rated (i.e., 0 = no and 1 = yes) across the 14 items which evaluated study design, selection bias, information bias, measurement bias, and confounding variables. A total percentage was calculated by dividing the number of items rated 1 by the number of applicable items and multiplying by 100. Quality ratings were categorised as poor (≤ 50%), fair (50.01–74.99%), and good (≥ 75%) as per previous studies (e.g., [55]).

### Meta-analysis

#### Main effect

The meta-analysis was run in JASP (version 0.18.3). A random effects model was used to estimate effect sizes between compulsive exercise and perfectionism [56]. The wald-type confidence interval calculated confidence intervals [18].

#### Assessment of heterogeneity

The Q-statistic with a *p*-value and I^2^ statistic were calculated to assess the heterogeneity of effect sizes [57]. A significant Q-statistic shows studies are heterogenous [58] and I^2^ indicates the proportion of variability that is attributable to between-study variance [58]. Heterogeneity of effect sizes was classified according to benchmark values, < 50%﻿, 50–75%, or ≥ 75, as low, moderate, and high respectively [59].

#### Moderator analyses

Study quality and gender were assessed in meta-regressions as potential sources of heterogeneity. Moderator analyses were only run if there was moderate to high heterogeneity and four or more studies in each subgroup [60].

#### Publication bias and sensitivity analyses

Egger’s test was used to assess for publication bias [61]. Sensitivity analyses were used to evaluate the influence of low-quality studies on the results (i.e., study quality rating of ≤ 50%; [55]).

## Results

### Study selection and characteristics

The search results and study inclusion procedure are detailed in Fig. 1. The final included sample was 7 cross-sectional studies, with a total sample size of 3,117 participants, with 49% female participants aged 12–65 years (*M* age = 21.78 years). Table 3 presents an overview of the characteristics of included studies. Three studies included samples of athletes or adults who exercised regularly, three studies included students, and one study included a clinical sample of adolescents diagnosed with eating disorders. In the one clinical study of Creswell et al. (13]; see Table 3) this was a clinical sample of 149 underweight adolescent girls who were outpatients (70%) and inpatients (30%) at a paediatric eating disorder treatment service. The adolescent clinical sample predominately had diagnoses of anorexia nervosa (AN; 66% AN restricting type; 21% unspecified feeding or eating disorder; 13% AN binge-eating/purging type).

### Study quality and risk of bias

Quality ratings of the studies are presented in Table 4, ordered from high to low risk of bias to distinguish the most trustworthy evidence. The quality of studies ranged from 45 to 66%, the average study rating was fair (*M* = 58.33%, *SD* = 8.74%). The cross-sectional study design was the main limitation as exposures are measured at the same time as outcomes, precluding causal inferences (Items 6 and 7; [54]). All included studies used reliable and valid measures contributing to higher overall quality.

### Meta-analysis results

#### Preliminary meta-analysis of the association between compulsive exercise and total perfectionism

**Fig. 2:**
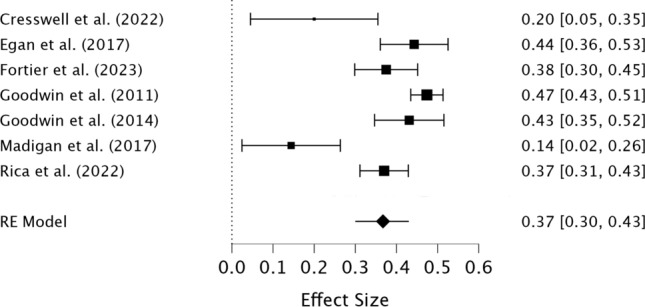
Forest plot of the correlations between total perfectionism and compulsive exercise

The preliminary meta-analysis of seven studies indicated a positive, moderate pooled association effect size between total perfectionism and compulsive exercise (*r* = 0.37, 95% CI [0.30, 0.43], *z* = 11.13, *p* < 0.001), suggesting that higher total perfectionism is associated with greater compulsive exercise (see Fig. 2). Heterogeneity was moderate (I^2^ = 80.22%) and significant (Q = 39.71, *p* < 0.001). The results of the meta-regression indicate that the quality of studies and percentage of female participants did not significantly influence the effect size (see Table 5). The level of heterogeneity remained high in the meta-regression (I^2^ = 76.75%).

Egger’s test was significant (*p* < 0.05), indicating that publication bias was likely an issue. Sensitivity analyses identified that after the removal of studies with a poor-quality rating (≤ 50%; *n* = 2), the association between perfectionism and compulsive exercise remained moderate (*r* = 0.41, 95% CI [0.36, 0.47], *z* = 14.62, *p* < 0.001). The level of heterogeneity decreased from high to moderate (I^2^ = 60.89%).

#### Preliminary meta-analysis of the association between compulsive exercise and perfectionistic strivings

**Fig. 3:**
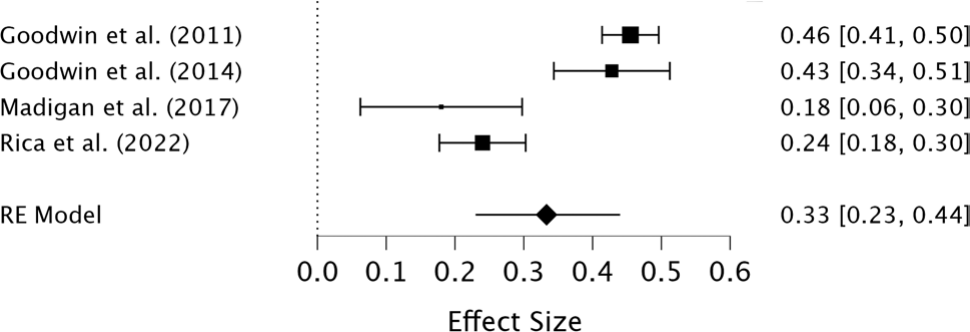
Forest plot of the correlations between perfectionistic strivings and compulsive exercise

The preliminary meta-analysis of the four studies indicated a positive, moderate pooled association effect size between perfectionistic strivings and compulsive exercise (*r* = 0.33, 95% CI [0.23, 0.44], *z* = 6.21, *p* < 0.001), suggesting that higher perfectionistic strivings is associated with greater compulsive exercise (see Fig. 3). Heterogeneity was high (I^2^ = 89.09%) and significant (Q = 44.25, *p* < 0.001). The results of the meta- regression indicate that the quality of studies and percentage of female participants did not significantly influence the effect size (see Table 5). The level of heterogeneity decreased from high to low in the meta-regression (I^2^ = 31.91%).

Egger’s test was not significant (*p* > 0.05), indicating that publication bias was unlikely an issue. Sensitivity analyses identified that after the removal of studies with a poor-quality rating (≤ 50%; *n* = 2), the association between perfectionistic strivings and compulsive exercise remained moderate (*r* = 0.45, 95% CI [0.41, 0.49], *z* = 23.84, *p* < 0.001). The level of heterogeneity decreased from high to nil.

#### Preliminary meta-analysis of the association between compulsive exercise and perfectionistic concerns

**Fig. 4:**
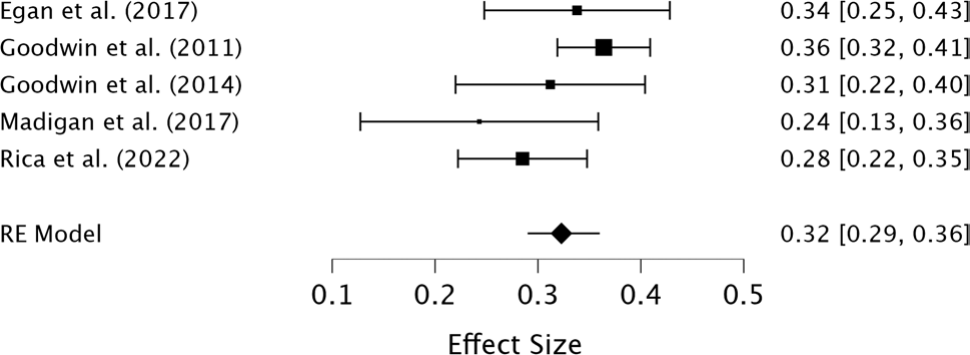
Forest plot of the correlations between perfectionistic concerns and compulsive exercise

The preliminary meta-analysis of the five studies indicated a positive, moderate pooled association effect size between perfectionistic concerns and compulsive exercise (*r* = 0.32, 95% CI [0.29, 0.36], *z* = 17.35, *p* < 0.001), suggesting that higher perfectionistic concerns is associated with greater compulsive exercise (see Fig. 4). Low heterogeneity was detected (I^2^ = 20.66%; Q = 6.49, *p* = 0.165). The results of the meta-regression indicate that the quality of studies and percentage of female participants did not significantly influence the effect size (see Table 5). No heterogeneity was detected (I^2^ = 0%).

Egger’s test was not significant (*p* > 0.05), indicating that publication bias was unlikely an issue. Sensitivity analyses identified that after the removal of studies with a poor-quality rating (≤ 50%; *n* = 2), the association between perfectionism and compulsive exercise remained moderate (*r* = 0.35, 95% CI [0.31, 0.39], *z* = 18.64, *p* < 0.001). Nil heterogeneity was detected (I^2^ = 0%).

## Discussion

This is the first meta-analysis to evaluate the association between perfectionism and compulsive exercise. It should be noted that given the small number of studies included, the conclusions are tentative and should be viewed as a preliminary attempt to provide a synthesis of the data, requiring a more robust meta-analysis when there are a greater number of studies available. The effect size was larger than hypothesised for the association between total perfectionism and compulsive exercise, and for the relationship between perfectionistic strivings and compulsive exercise, while it was as expected for the association between perfectionistic concerns and compulsive exercise; all effect sizes were positive and moderate. These findings provide partial indirect support for Meyer et al.’s [1] cognitive behavioural model of compulsive exercise where perfectionism maintains eating disorder symptoms indirectly through compulsive exercise.

Research suggests that inconsistency in conceptualisations of compulsive exercise may account for the wide range of prevalence rates reported in the literature [24] and may partially explain the higher than predicted relationship between compulsive exercise and total perfectionism. The CET, compared to other exercise measures, has demonstrated a stronger association with eating disorder symptoms [28, 29]. Given the CET was developed specifically for use in eating disorder research and assessment, and that perfectionism is one of the core components of the cognitive-behavioural model of compulsive exercise, this stronger relationship would be expected. Further, as perfectionism has been identified as one of the four maintaining factors in the transdiagnostic model of eating disorders [26], this may explain the stronger than expected relationship between constructs. While recent meta-analyses have confirmed perfectionism is related to greater eating disorder symptoms [18, 19], this is the first preliminary meta-analysis to quantify the relationship between perfectionism and compulsive exercise.

While there was considerable heterogeneity across studies, sensitivity analyses suggest this was primarily due to low study quality. Meta-regression analyses indicated that gender and study quality did not impact the strength of the association. The small number of available studies limits generalisations from these results. To make direct comparisons, future studies should investigate gender differences in the associations between perfectionistic strivings and concerns and compulsive exercise in the same sample to ensure efficacious treatment is developed for all genders [62].

### Clinical implications

Our results, combined with research showing that, if left untreated, compulsive exercise is associated with lower rates of remission in eating disorder patients, [63] indicate the need for compulsive exercise to be clinically assessed and targeted in the treatment of eating disorders [21]. Compulsive exercise is a transdiagnostic feature of eating disorders [63]. A recent large-scale study of over 7000 participants with a current or lifetime history of eating disorders, found that compulsive exercise was associated with higher eating disorder symptoms, depression, anxiety, obsessive–compulsive traits, and suicidal ideation [64]. Perfectionism, which is implicated as a transdiagnostic process [65], is similarly associated with higher eating disorder symptoms, anxiety, depression, and obsessive–compulsive symptoms [66]. The close relationship between compulsive exercise and perfectionism was shown to be indirectly mediated through eating disorder symptoms [21]. The results of the current meta-analysis are consistent with Adam et al.’s [21] findings that compulsive exercise is directly associated with eating disorder symptoms and perfectionism.

The findings of our review support the idea that treatments aimed at reducing compulsive exercise should be further evaluated. Valentine et al. [23] found that Cognitive Behaviour Therapy (CBT) for perfectionism was effective in reducing perfectionism, eating disorder symptoms, and compulsive exercise in individuals engaging in regular exercise. Similarly, other studies have shown treatment targeting compulsive exercise is effective in reducing compulsive exercise and eating disorder symptoms [22, 67]. Further, a recent treatment study comparing Compassion-Focused Therapy (CFT), and CBT showed both were effective in reducing compulsive exercise among adults with eating disorders [67]. Self-compassion has been suggested as a possible mechanism of change in eating disorder treatment [68]. Both perfectionistic strivings and perfectionistic concerns are negatively correlated with self-compassion [69], and higher perfectionism and lower self-compassion are associated with greater eating disorders in female adolescents [70]. Components of CFT are already within CBT for perfectionism [71], which has demonstrated efficacy in the reduction of symptoms of eating disorders [72, 73].

Interestingly CFT compared to CBT had a larger effect on the use of exercise as a maladaptive affect regulator [67]. Similarly CompuLsive Exercise Activity TheraPy (LEAP; [22]), which is a cognitive behavioural therapy treatment based on Meyer and colleagues’ cognitive behavioural model of compulsive exercise [1] also focuses on negative affect regulation [29]. In an RCT of LEAP therapy, the treatment was added to CBT-AN and the combined treatment with LEAP added was compared to standard CBT-AN. The combined treatment incorporating LEAP was shown to be as effective as standard CBT-AN in improving eating disorder symptoms, anxiety, depression, and health related quality of life [22]. Building on the suggestion by Harris et al. [29], future research should assess the comparative efficacy of CBT for perfectionism [23] and LeAP therapy [22], as well as CFT [67], in a clinical sample to determine which therapy is superior in reducing compulsive exercise and eating disorder symptoms.

### Strengths and limits

Despite agreement on the relevance of compulsive exercise in the aetiology of eating disorders [74], a primary limitation of the literature has been a lack of consensus on defining compulsive exercise, resulting in multiple measures of ‘problematic’ exercise based on differing underlying clinical and psychological constructs [24]. A key strength of this review was the inclusion of only the Compulsive Exercise Test [27]. This increases the validity of the findings of the current meta-analysis as the CET [27] is the most theoretically and clinically sound assessment tool [75]. Further, sensitivity analyses indicated no significant difference on the overall effect size based on study quality and showed low quality studies accounted for a substantial degree of heterogeneity found.

Several limitations should be noted. First, the number of studies included in the meta-analysis is low and this limits the robustness of the findings. Therefore, generalisations based on this preliminary meta-analysis should be tentative. Second, grey literature and manual searches were not completed, which may have also contributed to the publication bias and low number of studies. It is noted that selective publication may also be an issue. Third, only one study included a clinical sample. Given the CET was developed in the context of eating disorders, more studies are needed to investigate if clinical status is a significant moderator. Fourth, the studies included were cross-sectional in design, precluding any casual conclusions, which is an ongoing limitation recognised in the literature [76]. Future research should investigate this relationship in longitudinal group-level and intra-individual network analyses [76] to examine the inter-relationship between perfectionism, compulsive exercise, and eating disorder symptoms over time.

### Conclusion

This is the first meta-analysis examining the relationship between perfectionism and compulsive exercise. The preliminary meta-analysis showed total perfectionism, perfectionistic strivings, and perfectionistic concerns were all significantly moderately correlated with compulsive exercise, indicating higher perfectionism is related to greater compulsive exercise. Further research on compulsive exercise is needed to determine the best intervention approach given its relationship to perfectionism and relevance in the context of eating disorders.

#### What is already known on this subject?

Three prior systematic reviews have examined the association between perfectionism and exercise addiction using multiple different measures of problematic exercise with conflicting conclusions. This meta-analysis is the first to quantify the association between perfectionism and compulsive exercise, as measured by the Compulsive Exercise Test.

#### What this study adds?

This study quantified the association between multidimensional perfectionism and compulsive exercise; effect sizes for the relationship between compulsive exercise and total perfectionism, perfectionistic strivings, and perfectionistic concerns were positive and moderate. It is the first meta-analysis to date quantifying the association between perfectionism and compulsive exercise, which is important to understand given its relevance to eating disorders.

## Data Availability

No datasets were generated or analysed during the current study.

## Appendix

see Table 1, 2, 3, 4, 5

**Table 1:** Search Terms

Concept	Search terms
Perfectionism	Perfectionis*
Compulsive exercise	(problematic or morbid or addiction or compulsive or dependence or excessive or obligatory or unhealthy or driven or rigid or abuse or patholog*) ADJ2 (exercise or “physical activity” or training or running) or “compulsive exercise test” or overexercis* or over-exercis* or overtraining

*: truncation symbol; ADJ2: proximity operator for Ovid databases that specifies to retrieve records that contain search terms in any order within two words

**Table 2:** Scales MeasuringPperfectionism and the Classification of Subscales into Perfectionistic Strivings and Perfectionistic Concerns

Scale	Perfectionistic concerns	Perfectionistic strivings
Child and Adolescent Perfectionism Scale (CAPS; Flett et al., 2000)	Socially prescribed perfectionism	Self-oriented perfectionism
Multidimensional Perfectionism Scale (FMPS; Frost et al., 1990)	Concern over mistakes, doubts about actions, parental expectations, parental criticism	Personal standards, organisation
Multidimensional Perfectionism Scale Spanish version (FMPS; Frost et al., 1990; Carrasco et al., 2009)	Fear of mistakes, parental influence	Achievement expectations, organisation
Multidimensional Inventory of Perfectionism in Sport (MIPS; Stoeber et al., 2006)	Negative reactions to imperfection	Striving for perfection

Originally adapted from “The Relationship Between Perfectionism and Psychopathology: A Meta-Analysis,” by K. Limburg, H. J. Watson, M. S. Hagger, and S. J. Egan, 2016, *Journal of Clinical Psychology, 73*(10), p. 1303 (https://doi.org/10.1002/jclp.22435). Copyright 2016 by Wiley Periodicals.

**Table 3:** Study Characteristics and Summary of Included Studies

Authors	Country	Sample type	N^a^ (%F)	M age (age range)	Mean BMI [range]	Perfectionism Measure	CET
Cresswell et al. [13]	Australia	Female adolescents with ED	149 (100%)	14.90 (13–17)	− 1.95, [− 5.60 to − 1.32]	EDI-3-P	5 factor
Egan et al. [20]	Australia	Adults who exercised regularly	368 (50%)	32.24 (18–65)	NR	FMPS-CM; CPQ	3 factor
Fortier et al. [43]	Canada	Adolescent athletes	482 (53%)	14.98 (14–17)	NR	EDI-2-P	5 factor*
Goodwin et al. [77]	England	School Students	1488 (54%)	12.98 (12–14)	z = 0.08 (F) z = 0.32 (M)	CAPS	5 factor
Goodwin et al. [11]	England	School Students	369 (60%)	12.89 (12–14)	z = 0.28 (M) z = − 0.10 (F)	CAPS	5 factor
Madigan et al. [53]	England	Adult athletes	261 (26%)	20.9 (16–45)	NR	MIPS	3 factor
Rica et al. [78]	Spain	Male university students	850 (0%)	20.00	22.4	FMPS*	3 factor*

% F, percentage of sample that are female; BMI, body mass index; CAPS, Children and Adolescent Perfectionism Scale; CET, Compulsive Exercise Test; CPQ, Clinical Perfectionism Questionnaire; ED, eating disorder; EDI-2-P, Eating Disorder Inventory-2 Perfectionism subscale; EDI-3-P, Eating Disorder Inventory-3 Perfectionism subscale; F, female; FMPS, Frost Multidimensional Perfectionism Scale; FMPS-CM, Frost Multidimensional Perfectionism Concern over Mistakes subscale; M, male; MIPS, Multidimensional Inventory of Perfectionism in Sport; NR, not reported; z, Body Mass Index (BMI) values converted to age- and gender-adjusted z-score as reported in the primary studies as per recommendations for BMI reporting in children and adolescents [11]

^a^Total sample sizes for groups included in the meta-analysis

Asterisk denotes the scale has been translated

**Table 4:** Quality Assessment of Risk of Bias of Included Studies

Author (year)	1	2	3	4	5	6	7	8	9	10	11	12	13	14	Rating (%)
Cresswell et al. [13]	•	•	•	•				•	•		•	NA	NA	•	66.67
Fortier et al. [43]	•	•		•				•	•		•	NA	NA	•	66.67
Goodwin et al. [11]	•		•	•				•	•	•	•	NA	NR	•	66.67
Goodwin et al. [77]	•		•	•				•	•		•	NA	NA	•	58.33
Egan et al. [20]	•		NA	•				•	•		•	NA	NA	•	54.55
Rica et al. [78]	•		•					•	•		•	NA	NA	•	50.00
Madigan et al. [53]	•		NR					•	•		•	NA	NA	•	45.45

Study quality was assessed using the National Institute of Health Quality [54] Assessment Tool for Observational and Cross-sectional Studies

The overall score was categorised as poor (≤ 50%), fair (50.01–74.99%) and good (≥ 75%)

Bullet point denotes criteria was met

NR, not reported and NA, not applicable

**Table 5:** Summary Statistics for Meta-regression Analyses of Relationship between Perfectionism and Compulsive Exercise

	*k*	*B*	95% CI	I^2^ (%)	*p*
Total perfectionism				76.75	
Female participants (%)	7	− 0.21	− 0.57 to 0.15		0.25
Quality rating (%)	7	0.95	− 0.27 to 2.16		0.13
Perfectionistic strivings				31.91	
Female participants (%)	4	0.27	− 0.02 to 0.55		0.09
Quality rating (%)	4	0.57	− 0.45 to 1.59		0.27
Perfectionistic concerns				0.00	
Female participants (%)	5	0.12	− 0.10 to 0.34		0.26
Quality rating (%)	5	0.04	− 0.83 to 0.91		0.93

CI, confidence interval.
